# A Comparative Analysis of the Gene Expression Profiles of Small Cell Esophageal Carcinoma, Small Cell Lung Cancer, and Esophageal Adeno/Squamous Carcinoma

**DOI:** 10.3389/fsurg.2021.655159

**Published:** 2021-07-30

**Authors:** Di Liu, Junmiao Wen, Jiayan Chen, Boyan Wang, Xinyan Xu, Zhen Zhang, Min Fan

**Affiliations:** ^1^Department of Radiation Oncology, Fudan University Shanghai Cancer Center, Shanghai, China; ^2^Department of Oncology, Shanghai Medical College, Fudan University, Shanghai, China; ^3^Shanghai Key Laboratory of Radiation Oncology, Shanghai, China

**Keywords:** small cell esophageal carcinoma, small cell lung cancer, esophageal squamous carcinoma, gene expression profile, esophageal adenocarcinoma

## Abstract

**Purpose/objectives:** Primary small cell esophageal carcinoma (SCEC) is a rare malignancy without an established treatment strategy. This study investigated the gene expression profile of SCEC and compared it with the expression profiles of small cell lung cancer (SCLC) and esophageal adeno/squamous carcinoma (EAC/ESCC).

**Materials/methods:** All patients with SCEC, SCLC, and EAC/ESCC in the Surveillance, Epidemiology, and End Results (SEER) database 1973–2014 were included. Overall survival (OS) and prognostic analysis were conducted. *De novo* expression array analysis was performed on three pairs of frozen primary SCEC tissues and the corresponding normal samples from the institutional tissue bank using the Affymetrix HG U133 plus 2.0 Array. These data were complemented with public domain expression data sets from the Gene Expression Omnibus (GEO) repository using the same working platforms, which included primary SCLC, EAC/ESCC, and normal lung/esophagus specimens (series GSE30219 and GSE26886). After individual normalization, the primary tumors were submitted to statistical analysis (GeneSpring GX 13.0) to identify the differentially expressed genes (DEGs) relative to their paired normal tissues. Enrichments of genes categorized by function and gene interactions were analyzed by DAVID 6.8 and STRING 11.0, respectively.

**Results:** The clinical outcomes of the patients with SCEC were significantly more worse than those with EAC/ESCC and SCLC in the SEER database. SCEC had more DEGs in common with SCLC than EAC/ESCC [829 vs. 450; false discovery rate (FDR) < 0.01; and fold change ≥2], leading to a stronger correlation between SCEC and SCLC (Pearson's correlation coefficient was 0.60 for SCEC vs. SCLC, 0.51 or 0.45 for SCEC vs. ESCC or EAC, and the coefficient was 0.73 for ESCC vs. EAC). Similar findings were obtained by principal component analysis (PCA) using all DEGs retrieved from these four groups. Functional annotation showed that a higher proportion of pathways and biological processes were common between SCEC and SCLC and were associated with the cell cycle (mitosis), DNA replication, telomere maintenance, DNA repair, and P53 and RB pathways (Benjamini *p* < 0.05). Compared with EAC/ESCC, SCEC shared more co-upregulated DEGs coding for the aforementioned common pathways with SCLC (584 vs. 155). In addition, SCEC and SCLC were found to have possessed overlapping gene-interactive networks, with centromere protein F (*CENPF)*, never in mitosis gene A-related kinase 2 **(***NEK2)*, kinesin family member 11 (*KIF11)*, thymopoietin (*TMPO)*, and forkhead box protein M1 (*FOXM1)* as common skeletons centered by gene regulatory network (*NUF2)*.

**Conclusions:** This study is the first attempt to examine the genomic signatures of SCEC at the transcriptomic level and compare the expression profiles between SCEC, SCLC, and EAC/ESCC. Our preliminary data indicate that SCEC and SCLC display notably similar patterns of gene expression for mitosis and DNA repair. Further validation studies are warranted.

## Introduction

Small cell carcinoma (SCC) is a highly aggressive malignancy that predominantly arises in the lung. Primary small cell esophageal carcinoma (SCEC) is the most common extrapulmonary SCC (~2%), with a reported incidence rate of 0.05–3.1% among all esophageal neoplasms (1–3). Due to a lack of prospective clinical trials or cell line experimental data, a consensus on treatment strategies for patients with SCEC has not been reached (4, 5). Previous studies have indicated similarities in pathology and clinical manifestations between SCEC and small cell lung cancer (SCLC), and patients with SCEC are staged and treated following the well-established therapeutic strategies for SCLC (4, 6) However, patients with SCEC have a significantly worse prognosis than those with esophageal adeno/squamous carcinoma (EAC/ESCC) and SCLC. Generally, patients with SCEC die within 2 years of diagnosis and experience a median survival of only 8–13 months. Chemotherapy is initially effective for SCEC, but most patients suffer a rapid recurrence and respond inadequately to second-line chemotherapy. More effective and precise therapeutic strategies for SCEC are urgently needed (7–9).

The lung and esophagus arise from the anterior foregut endoderm in the thorax, and they share common properties during development (10, 11). Theoretically, on one hand, SCCs in the lung and esophagus may be more similar than those occurring in other organs. On the other hand, the tissue of origin of a tumor is just as important as the mutations that drive it. The tissue of origin is an important determinant of how a tumor meets its metabolic needs (12). Thus, it seems essential to analyze the molecular characteristics of SCEC and identify the differences between SCLC and EAC/ESCC.

Although the genetic landscape of SCLC and EAC/ESCC has been extensively studied, little is known about SCEC (13–15). Gene expression profiling can investigate altered cellular mechanisms, thus improving our understanding of various diseases and enabling the development of novel therapeutic targets (16). SCEC, SCLC, and EAC/ESCC are highly aggressive cancers, but their detailed differences on the transcriptional levels are currently unknown. To the best of our knowledge, comparative analyses of gene expression profiles of these malignancies have not been reported so far, which is the starting point of this study.

In this study, we compared the overall survival (OS) data of SCEC, SCLC, and EAC/ESCC from the Surveillance, Epidemiology, and End Results (SEER) database. Then, genes with significantly altered expression in SCEC were screened and identified. We compared the gene expression profile of SCEC with the known data of SCLC and EAC/ESCC to highlight biomolecular markers with potential clinical significance. Finally, quantitative reverse transcription (qRT)-PCR analysis was performed to confirm the differential expression of 10 of these genes.

## Materials and Methods

### Patient Collection

This study utilized the SEER-18 registry databases, which currently cover 28% of the population of the United States. SEER routinely collects demographic, tumor site, stage at diagnosis, the first course of treatment, and follow-up of vital status data. We retrieved data from 1973 to 2014 using SEER 8.3.5 software and searched for all cases of SCEC using the ICD-O-3 codes 8041, 8043, and the primary site codes C150-159. In addition, patients who were diagnosed with other subtypes of esophageal neoplasms during the same period were also identified according to the corresponding ICD-O-3 codes (adenocarcinoma: 8050, 8140-8147, 8160-8162, 8180-8221, 8250-8507, 8514, 8520-8551, 8560, 8570-8574, 8576, and 8940-8941; squamous cell carcinoma: 8070-8078, 8083, and 8084). Patients with SCLC were identified using the primary site codes C340-349 and the ICD-O-3 codes 8041 and 8043. Patients were deemed eligible if they were ≥18 years old, had more than 1 month of follow-up time, and the first primary tumor.

### Statistical Analysis

Our analysis included age at diagnosis, sex, race, SEER summary stage, marital status, months of survival, and vital status. A log-rank test was conducted to compare the Kaplan–Meier survival curves. Overall survival (OS) was measured from the date of the initial treatment to the date of death or the last day of follow-up. Multivariate analyses with the Cox proportional hazards model were performed to evaluate the covariate effect on OS. Hazard ratios with 95% CIs were employed to quantify the strength of the association between the predictors and survival. A two-tailed *p*-value of < 0.05 was considered statistically significant. All statistical calculations using were performed R software version 3.4.2 (Institute for Statistics and Mathematics, Vienna, Austria; www.r-project.org).

### Tissues and Total RNA Preparation

A total of three SCEC tissues and matched adjacent non-cancerous tissues were dissected from the surgical specimens and reviewed by at least two independent expert pathologists, and the diagnosis of SCEC was confirmed by H & E staining and immunohistochemistry (IHC) for synaptophysin, chromogranin A, neuro-specific enolase (NSE), neural cell adhesion molecule (CD56), and antigen KI-67 (Ki67). Any sample with squamous or adenocarcinoma differentiation was excluded. These tumor samples were pathologically assessed to have a purity of at least 60% and minimal necrosis. Additionally, by pathological assessment adjacent non-tumorigenic tissue was confirmed to be free of tumor contaminants. The selected patients did not receive any anticancer therapy before surgery and had not been diagnosed with any other cancer. Ethics approval for this study was granted by the Human Research Ethics Committee of Fudan University Shanghai Cancer Center (FUSCC), and informed consent was obtained from all patients. Two of the patients were women, and the patients had a median age of 59 years (range from 56 to 67). The primary location of all of the tumors was the middle thoracic region of the esophagus and was stage III (TNM staging system of the American Joint Committee on Cancer, 6th edition) or limited stage (Veteran's Administration Lung Cancer Study Group, VALSG). All of the patients were deceased at the last follow-up.

Total RNA was extracted from the SCEC and matched adjacent non-cancerous tissues with TRIzol reagent (Life Technologies, Carlsbad, CA, USA) according to the instructions of the manufacturer. The concentration and purity of the RNA in each sample were determined by measuring the absorbance at 260 and 280 nm. RNA integrity was confirmed by electrophoresis on 1% agarose gels. Only RNA samples with a renewable identification number (RIN) > 7.5 were applied in later microarray and quantitative reverse transcription (qRT) -PCR experiments.

### Gene Expression Microarray and Interactive Analysis

The generation of cDNA and cRNA, hybridization with Affymetrix HG U133 Plus 2.0 Array (Affymetrix, Santa Clara, CA), scanning, and microarray gene expression data analyses were performed as previously described (4). These data were complemented with public domain expression data sets from the GEO repository using the same platforms, which included primary SCLC, primary EAC/ESCC, and normal lung/esophagus specimens (series GSE30219 and GSE26886). The quality control of the samples was assessed by boxplots and principal component analysis (PCA) (Supplementary Figure 1). A pairwise comparison was performed by direct comparison of differentially expressed genes (DEGs) filtered from the above four paired groups (SCEC, SCLC, EAC, and ESCC), starting from the raw data (CEL files), after individual normalization within each paired group and applying the same analytical approach using GeneSpring GX 13.0 software. The DEGs were analyzed through moderated *t*-test analysis with Benjamini–Hochberg multiple testing correction using the following parameters: fold change (FC) ≥2 and false discovery rate (FDR) cutoff < 0.01. The DEGs were visualized in a volcano plot (Supplementary Figure 2). Gene enrichments with functional annotation and gene interaction networks were analyzed by DAVID 6.8 and STRING 11.0, respectively.

### Validation of Microarray by qRT-PCR

Ten genes differentially expressed in SCEC compared wit h matched adjacent non-cancerous tissues identified in the microarray experiment were selected for validation by qRT-PCR. Total RNA extraction, cDNA synthesis, and quantification of gene expression levels were performed on a 7,500 Fast Real-Time PCR cycler (Applied Biosystems, Foster City, CA) with SYBR Green reagents (Takara Bio Inc, Shiga, Japan). as previously described (4). Primers were designed and synthesized by BioTNT Co. (Shanghai, China), and their sequences are listed in Supplementary Table 1. β-actin was used as an endogenous control. PCR reactions of each sample were conducted in triplicate. The relative expression of the target genes was calculated by 2^−Δ*ΔCt*^.

## Results

### SEER Data of SCEC as Compared to SCLC and EAC/ESCC

Surveillance, Epidemiology, and End Results data of SCEC as compared to SCLC and EAC/ESCC.

**Figure 1:**
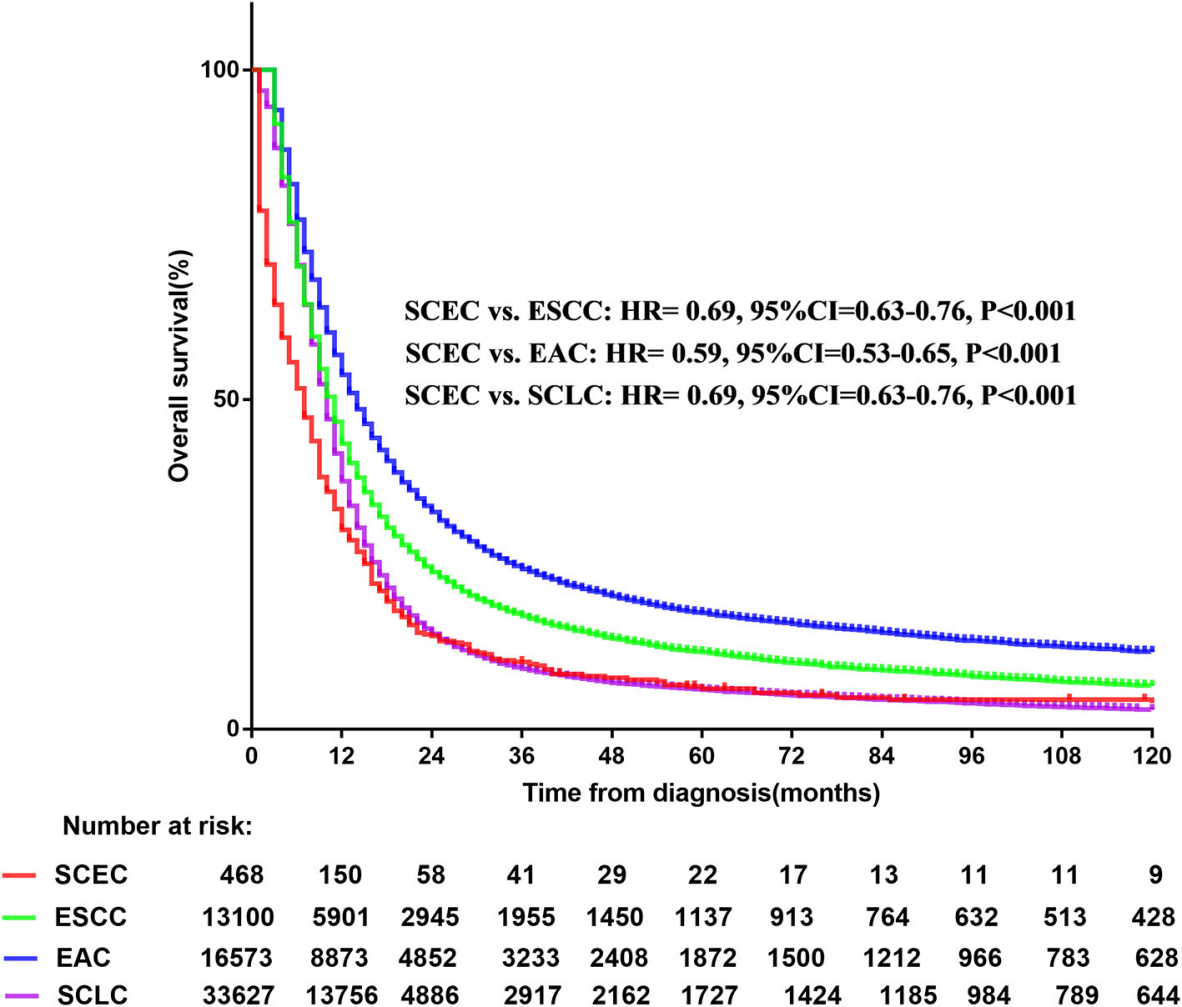
Overall survival comparison for SCEC, ESCC, EAC, and SCLC patients. SCEC, Small cell esophageal cancer; ESCC, esophageal squamous cell cancer; EAC, esophageal adenocarcinoma; SCLC, small cell lung cancer; HR, hazard ratio; CI, confidence interval.

**Figure 2:**
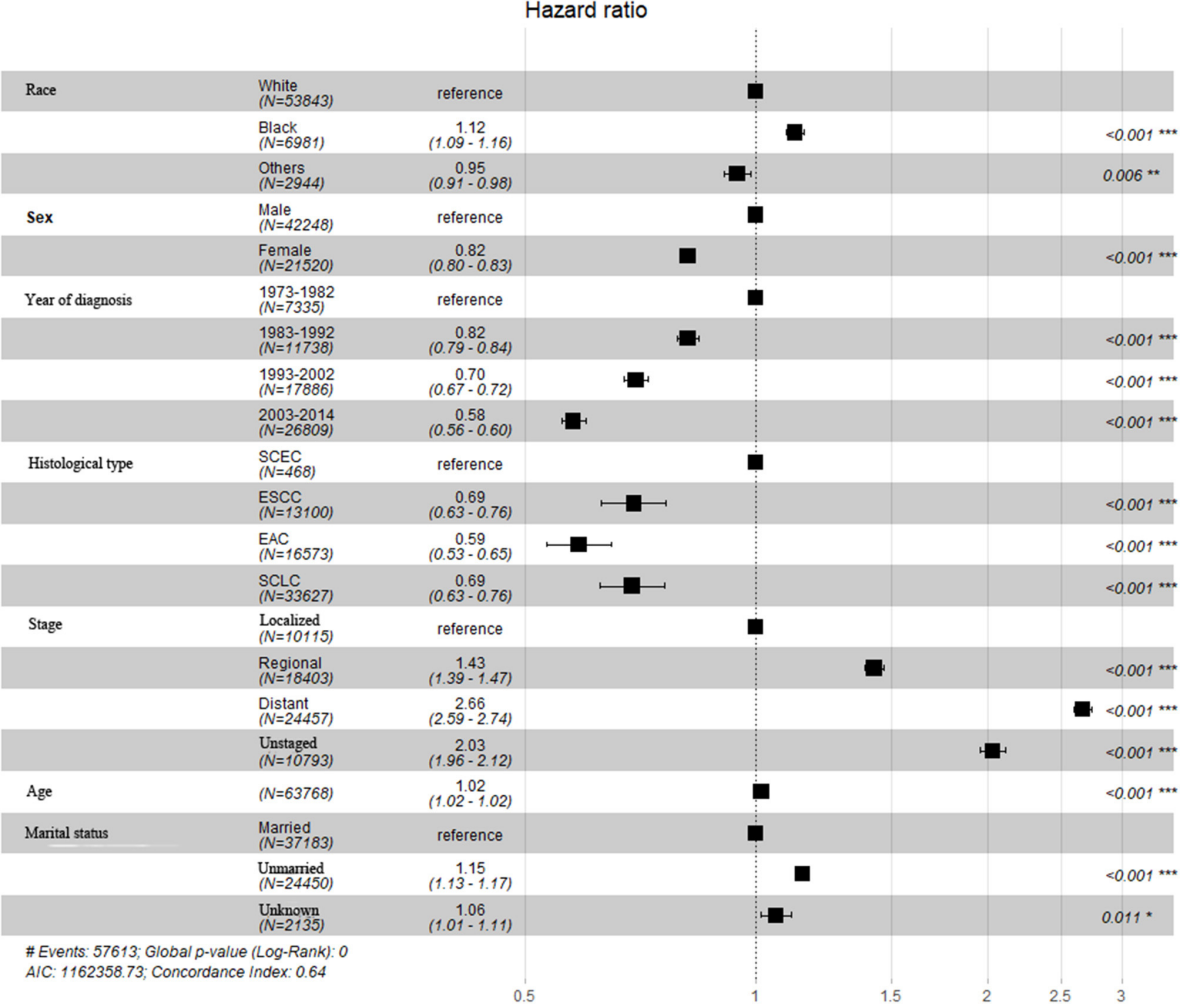
Multivariate and Cox regression analysis for SCEC, ESCC, EAC, and SCLC patients.

A total of 63,768 patients diagnosed from 1973 to 2014 were identified from the SEER database. Among them, patients with SCLC accounted for the largest proportion (33,627, 52.7%), followed by EAC (16,573, 26.0%), ESCC (13,100, 20.5%), and SCEC (468, 0.7%). The baseline characteristics are summarized in Table 1. Kaplan–Meier analyses and log-rank testing were conducted to compare the OS among these specific histological types, and the results are shown in Figure 1. Regarding OS, the 5-year survival for patients with SCEC was 6.1%, similar to that of patients with SCLC (5.9%). Patients with EAC (5-year OS: 17.6%) and ESCC (5-year OS: 11.6%) had a better prognosis than those with the other two types. To further refine the analysis on the prognostic value of histological types, we utilized Cox models to predict OS incorporating age at diagnosis, sex, ethnicity, year of diagnosis, SEER summary stage, and marital status and found that the prognosis of patients with SCEC was significantly inferior to that of the other three histological types (*p* < 0.001, Figure 2).

**Table 1:** Basic characteristics of SCEC, ESCC, EAC, and SCLC in the SEER database.

**Stratified by histology type**	**level**	**SCEC**	**ESCC**	**EAC**	**SCLC**	***p*-value**
*n*		468	13,100	16,573	33,627	
Race (%)	White	361 (77.1)	8,160 (62.3)	15,785 (95.2)	29,537 (87.8)	< 0.001
	Black	78 (16.7)	3,642 (27.8)	386 (2.3)	2,875 (8.5)	
	Others	29 (6.2)	1,298 (9.9)	402 (2.4)	1,215 (3.6)	
Sex (%)	Male	282 (60.3)	8,816 (67.3)	14,515 (87.6)	18,635 (55.4)	<0.001
	Female	186 (39.7)	4,284 (32.7)	2,058 (12.4)	14,992 (44.6)	
Year at diagnosis (%)	1973–1982	43 (9.2)	1,622 (12.4)	271 (1.6)	5,399 (16.1)	<0.001
	1983–1992	67 (14.3)	2,204 (16.8)	997 (6.0)	8,470 (25.2)	
	1993–2002	115 (24.6)	4,608 (35.2)	4,003 (24.2)	9,160 (27.2)	
	2003–2014	243 (51.9)	4,666 (35.6)	11,302 (68.2)	10,598 (31.5)	
Stage (%)	Localized	81 (17.3)	4,040 (30.8)	4,078 (24.6)	1,916 (5.7)	<0.001
	Regional	78 (16.7)	5,133 (39.2)	6,362 (38.4)	6,830 (20.3)	
	Distant	240 (51.3)	3,927 (30.0)	6,133 (37.0)	14,157 (42.1)	
	Unstage	69 (14.7)	0 (0.0)	0 (0.0)	10,724 (31.9)	
Age at diagnosis [mean (SD)]		68.35 (11.97)	65.06 (11.01)	64.15 (11.60)	64.66 (9.94)	<0.001
Marital status (%)	Married	250 (53.4)	6,489 (49.5)	10,787 (65.1)	19,657 (58.5)	<0.001
	Unmarried	197 (42.1)	6,114 (46.7)	5,232 (31.6)	12,907 (38.4)	
	Unknown	21 (4.5)	497 (3.8)	554 (3.3)	1,063 (3.2)	

*SEER, Surveillance, Epidemiology, and End Results; SCEC, small cell esophageal carcinoma; ESCC, esophageal squamous carcinoma; EAC, esophageal adenocarcinoma; SCLC, small cell lung cancer; SD, standard deviation*.

#### Gene Expression Profile of SCEC Compared to SCLC and EAC/ESCC

A total of 1,485 DEGs in SCEC vs. adjacent non-cancerous tissues with 879 upregulated genes and 606 downregulated genes were identified in a previous study; these were enriched for overexpression of proliferation-associated and neuroendocrine-associated genes (4). Pathway analysis showed enrichment of DNA replication, cell cycle, mitosis, telomere maintenance, DNA repair, and p53 and RB pathways by the database for annotation, visualization, and integrated discovery (DAVID) annotation (count ≥10 and Benjamini *p*-value < 0.01).

**Figure 3:**
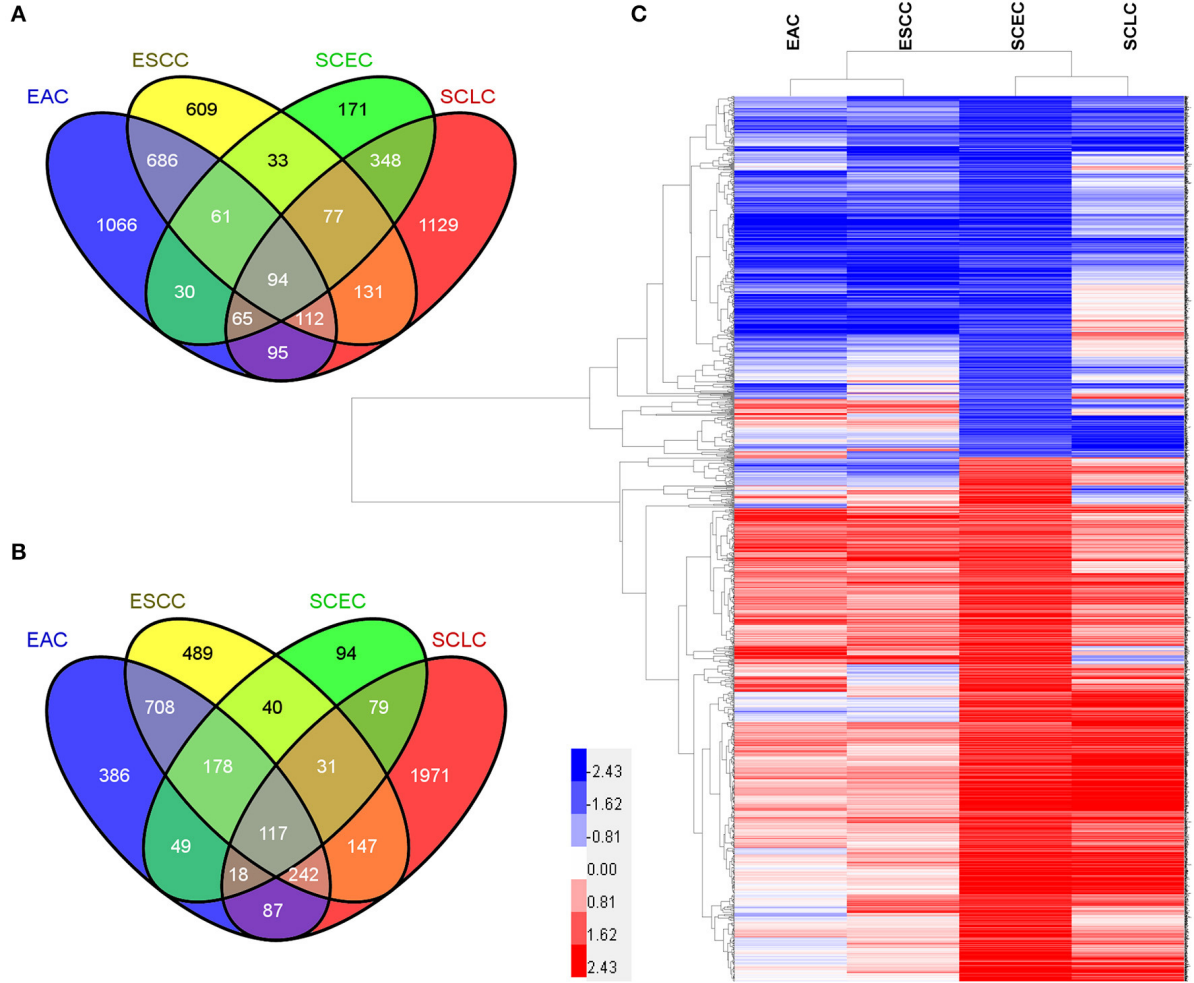
Differential expression analysis in SCEC, SCLC, EAC, and ESCC groups. **(A)** Venn diagram showing the number of DEGs in pairwise comparisons among groups of samples. **(B)** Pearson's correlation matrix indicated that SCEC proved to be more correlated to SCLC than EAC/ESCC. **(C)** Principal Component Analysis (PCA) showing the relationships between the groups of samples that were compared.

**Figure 4:**
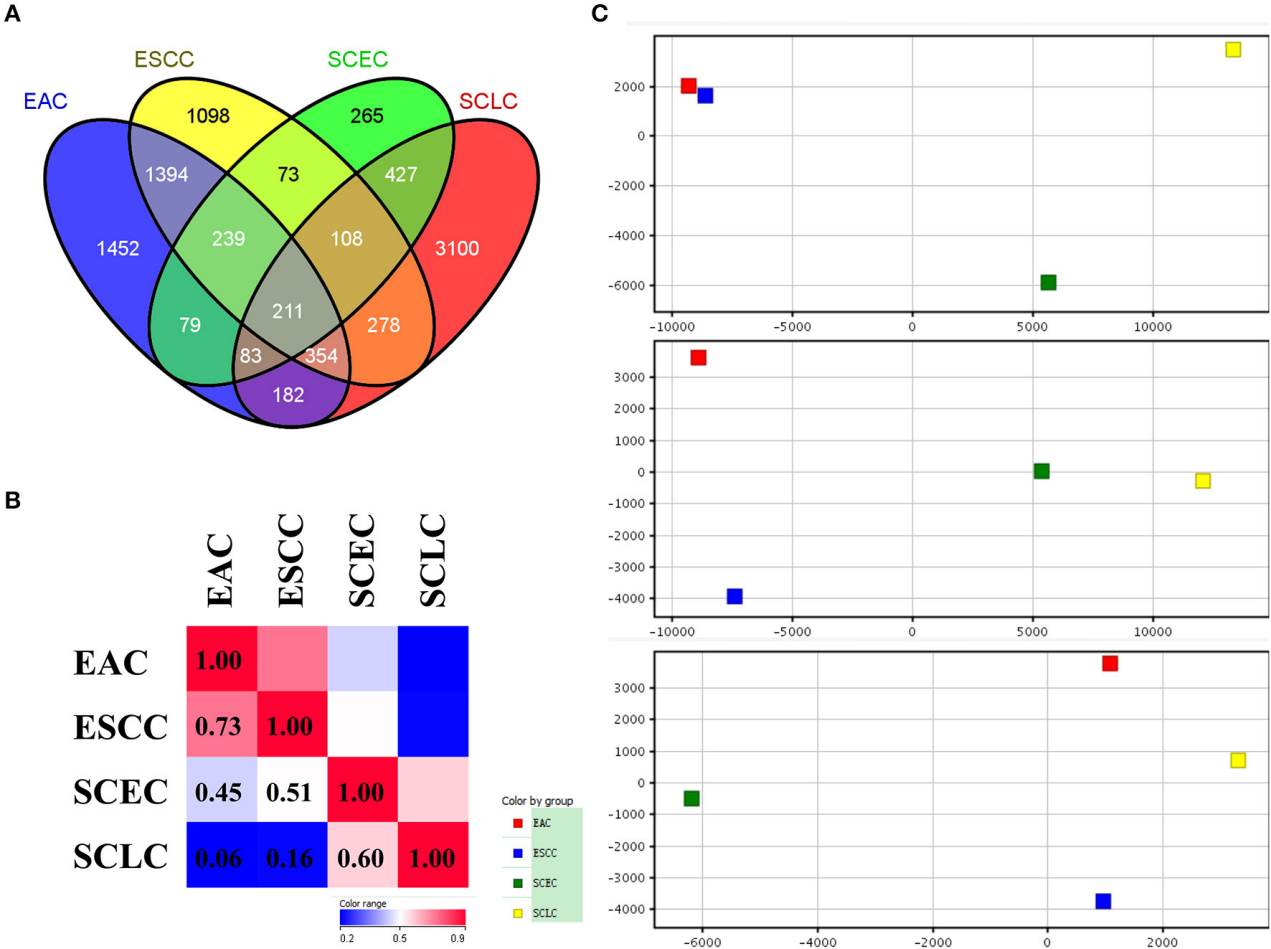
Differential expression analysis in SCEC, SCLC, EAC, and ESCC groups divided by up-regulated and down-regulated genes. **(A,B)** Venn diagram showing SCEC shared more co-up regulated DEGs with SCLC compared with EAC/ESCC. **(C)** Hierarchical clustering of SCEC, SCLC, EAC, and ESCC groups. The color scale represents the level of expression from low (blue) to high (red).

The expression data demonstrated that SCEC had more DEGs in common with SCLC than EAC/ESCC (829 vs. 450; FDR < 0.01; and FC ≥2; Figure 3), leading to a stronger correlation between SCEC and SCLC (Pearson's correlation coefficient was 0.60 for SCEC vs. SCLC, 0.51 or 0.45 for SCEC vs. ESCC or EAC, and 0.73 for ESCC vs. EAC). Similar findings were obtained by PCA using all DEGs retrieved from these four groups (Figure 4). Functional annotation showed that a higher proportion of biological processes or pathways were shared in common between SCEC and SCLC and were associated with the cell cycle, mitosis, DNA replication, telomere maintenance, DNA repair, and p53 and RB pathways (count ≥10 and Benjamini *p-*value < 0.05; Table 2 and Supplementary Tables 2–4). Compared with EAC/ESCC, SCEC shared more co-upregulated DEGs coding for the aforementioned common pathways with SCLC (584 vs. 155; Figure 3). Hierarchical clustering of SCEC, SCLC, and EAC/ESCC according to gene ontology (GO) annotation is shown in Supplementary Figure 3. In addition, SCEC and SCLC possessed overlapping gene-interactive network with *CENPF, NEK2, KIF11, TMPO*, and *FOXM1* as common skeletons centered by *NUF2* (Supplementary Figure 4). The genes involved in the SCEC-regulated network were related to cell cycle, mitosis, cell cycle checkpoint, spindle organization, microtubule binding, cytoskeletal protein binding, and other biological processes (Supplementary Tables 6, 7).

**Table 2:** DAVID annotation of DEGs in SCEC group.

**Database**	**Name**	**Count^**a**^**	**Benjamini *p*-value**
KEGG	**DNA replication** ^**b**^	19	8.88E-10
	**Cell cycle**	33	8.61E-09
	**P53 signaling pathway**	19	4.80E-05
	Progesterone-mediated oocyte maturation	18	0.00457
	Base excision repair	11	0.00419
	Oocyte meiosis	20	0.00790
REACTOME	**Cell cycle, mitotic**	100	3.47E-36
	**DNA replication**	29	7.19E-08
	**DNA repair**	22	0.00173
	Cell cycle checkpoints	23	0.00184
	**Telomere maintenance**	14	0.00713
PANTHER	**P53 pathway**	22	0.0456
GO BP (TOP10)	**M phase**	99	3.91E-28
	**M phase of mitotic cell cycle**	78	1.97E-26
	**Mitosis**	77	2.12E-26
	**DNA replication**	61	2.41E-18
	**DNA metabolic process**	98	2.54E-13
	Mitotic sister chromatid segregation	18	1.73E-07
	Spindle organization	20	1.55E-07
	Cell cycle checkpoint	27	2.09E-06
	Regulation of cell cycle process	28	6.97E-05
	**DNA repair**	50	7.34E-05
GO MF	**Pyrophosphatase activity**	91	0.00138
	**Adenyl ribonucleotide binding**	152	0.00573
	**Guanyl ribonucleotide binding**	44	0.0486

a *Threshold values: count ≥10 and Benjamini p-value < 0.01.*

b *The biological processes or pathways in common between SCEC and SCLC were in bold.*

*GO, gene ontology; BP, biological process; MF,molecular function*.

#### Validation of Microarray Results by qRT-PCR

**Figure 5:**
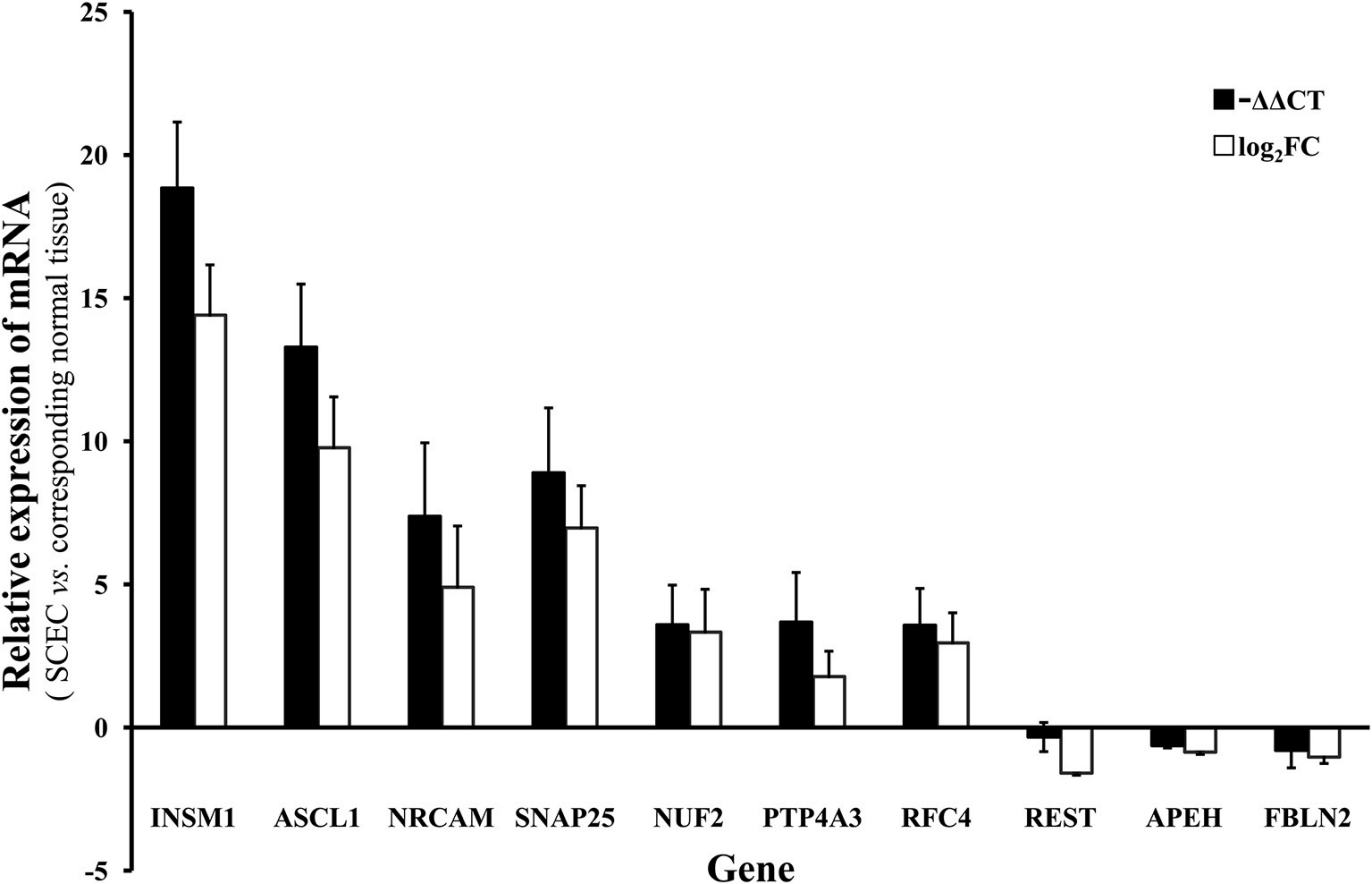
The mRNA level of of *INSM1, ASCL1, NRCAM, SNAP25, NUF2, PTP4A3, RFC4, REST, APEH*, and *FBLN2* in SCEC. Expression levels in SCEC were compared with the corresponding normal tissues. The *X* axis display gene symbols and the *Y* axis shows gene expression log ratios from microarray or qRT-PCR. Bars: standard error (SE).

Genes of interest identified by microarray were validated by qRT-PCR. The genes assayed were neuroendocrine-associated genes (*INSM1, ASCL1, NRCAM*, and *SNAP25*), one gene centered in the gene regulatory network (*NUF2*), and five possibly cancer-associated genes (*PTP4A3, RFC4, REST, APEH*, and *FBLN2*). The microarray and the qRT-PCR results demonstrated that *INSM1, ASCL1, NRCAM, SNAP25, NUF2, PTP4A3*, and *RFC4* were significantly upregulated while *REST, APEH*, and *FBLN2* were downregulated (Figure 5).

## Discussion

Small cell carcinoma is a high-grade neoplasm characterized by markers of neuroendocrine differentiation and aggressive histological features (high mitotic rate, extensive necrosis, and nuclear atypia), which confers a poor clinical prognosis (5, 17, 18). The majority of SCCs originate within the lung followed by the esophagus (3, 19–21). SCEC is a very rare disease with a tendency to metastasize early through lymph and blood circulation, and many recommendations about the treatment approach to SCEC are extrapolated from research on SCLC. Treatments for SCEC include surgical resection, chemotherapy, radiotherapy, and combinations of these treatments. First-line systemic chemotherapy with a platinum agent (cisplatin or carboplatin) and etoposide is recommended for most patients; however, response durations are often short, and long-time survivors are rare (22–24). Therefore, SCLC treatments are not sufficient or optimal for patients with SCEC. In addition, SCC originating in different organs may be distinct, as suggested in the literature study (20).

This study compared the survival data in the SEER database. Kaplan–Meier analysis showed that patients with SCEC had the worse OS, which was closer to SCLC and far worse than patients with EAC/ESCC. The multivariate analysis demonstrated that SCC was associated with a poor prognosis compared with pathological subtypes of squamous cell carcinoma or adenocarcinoma. Previous limited retrospective studies have suggested that SCEC is more malignant than other types of esophageal cancers (5). This study is in accordance with the literature study and is the first real-world study comparing the prognosis among SCEC with SCLC and EAC/ESCC using data from a large dataset.

The histogenesis of SCEC is controversial, and no definite conclusions have been made. It is assumed that SCEC may arise from amine precursor uptake and decarboxylation (APUD) cells or multipotent reserve cells (25, 26). SCEC and SCLC share several histological features, which support the theory that SCC arises from APUD cells. Observations of heterogeneous carcinoma components, including EAC/ESCC or mucoepidermoid carcinoma and ESCC *in situ*, provide evidence of derivation from multipotent reserve cells (27). It is interesting to elucidate the relationship of SCEC with SCLC and EAC/ESCC.

Genome sequencing studies have revealed several potential driver events in two other major subtypes of esophageal carcinoma and showed that they have distinct molecular characteristics, indicating the heterogeneity of esophageal carcinomas (13). A recently published SCEC landscape revealed the characteristics of the SCEC mutation spectrum and copy number variation spectrum, indicating that SCEC is highly distinct and may have a special genetic background (7). To date, detailed whole genetic studies of this disease at the mRNA level have been sparse.

In this study, we performed gene expression profiling of three patients with SCEC compared with matched adjacent non-cancerous tissues by microarray analysis. This study found that phosphatase and tensin homolog *(PTEN)*-, retinoblastoma protein (*RB)*-, and wingless and int-1 (*WNT)*-related gene sets and neuroendocrine- and proliferation-associated genes were significantly upregulated, while notch homolog 1 (*NOTCH)*-related gene sets were downregulated in SCEC, as previously described (4). Combined with the genomic aberrance of SCEC as reported previously (4, 7), the aforementioned gene sets and pathways might contribute to tumorigenesis and the development of SCEC, and these results were also in line with a recent publication (21).

Furthermore, we compared the gene expression profiles of SCEC between SCLC and EAC/ESCC. Our data demonstrated that there are more gene expression similarities between SCEC and SCLC than there are between SCEC and EAC/ESCC. We observed that DEGs in SCEC were significantly enriched in the cell cycle, mitosis, DNA replication, telomere maintenance, DNA repair, and p53 and RB pathways, which is highly concordant with those in SCLC. In addition, SCEC and SCLC display notably similar patterns of gene-interactive networks with *CENPF, NEK2, KIF11, TMPO*, and *FOXM1* as common skeletons centered by *NUF2*. In terms of the gene expression profile, the characteristics of SCEC are unique but more closely resembled SCLC than EAC/ESCC, as they share similar signaling pathways and gene-interactive networks. This similarity of expression profiles between SCEC and SCLC is consistent with the poor prognosis of SCC, since SCEC and SCLC are both highly aggressive.

With the deepening research studies into tumor biology, SCLC has entered the era of precision medicine (28). SCEC still remains outside the realm of precision medicine, where chemotherapy is the bedrock of treatment. Without biomarkers predictive of efficacy and toxicity and in the absence of precise identification of optimal treatment strategies, the prognosis of patients with SCEC is dismal. In addition, as our data suggested in the study, SCEC is a highly heterogeneous disease; however, its heterogeneous biology is poorly understood. Our attempt is only the first step, which has enabled a more comprehensive understanding of the transcriptomic landscape of SCEC.

A large-scale study is needed because our study had many limitations, such as a small number of samples and difficulty examining the protein level of interesting DEGs from microarrays. Only a small proportion of SCEC are resectable; inevitably, small numbers of samples are available. SCEC is a rare and deadly cancer. Although, we only examined insufficient cases, this study has added to the knowledge of SCEC at the transcriptomic level and highlights the potential useful genes and pathways for more precise diagnosis and treatment. Further, investigations based on the large-scale collection of samples are needed.

## Conclusions

This study is the first to examine the genomic signatures of SCEC from a gene expression perspective with comparison to SCLC and EAC/ESCC. SCEC has an extremely poor prognosis compared with SCLC and EAC/ESCC. Our preliminary data indicated that SCEC is a distinct disease and should be treated individually and precisely. Further, validation studies are warranted.

## Data Availability Statement

The datasets presented in this study can be found in online repositories. The names of the repository/repositories and accession number(s) can be found in the article/Supplementary Material.

## Ethics Statement

Written informed consent was obtained from the individual(s) for the publication of any potentially identifiable images or data included in this article.

## Author Contributions

Conception and design: DL, ZZ, and MF. Collection and assembly of data: JW, BW, and JC. Data analysis and interpretation: DL, JW, and XX. All authors contributed to manuscript writing, final approval of manuscript, and accountable for all aspects of the work.

## Conflict of Interest

The authors declare that the research was conducted in the absence of any commercial or financial relationships that could be construed as a potential conflict of interest.

## Publisher's Note

All claims expressed in this article are solely those of the authors and do not necessarily represent those of their affiliated organizations, or those of the publisher, the editors and the reviewers. Any product that may be evaluated in this article, or claim that may be made by its manufacturer, is not guaranteed or endorsed by the publisher.
