# Supplementary material for: A Comparative Analysis of the Gene Expression Profiles of Small Cell Esophageal Carcinoma, Small Cell Lung Cancer, and Esophageal Adeno/Squamous Carcinoma
Source: Front Surg. 2021 Jul 30;8:655159. doi: 10.3389/fsurg.2021.655159 (PMC8362887; doi:10.3389/fsurg.2021.655159)
Supplement: Supplementary file 1 [file Data_Sheet_1.DOC]

**SUPPLEMENTARY INFORMATION**

**LISTS OF FIGURES**

1 Box plot and Principal Component Analysis (PCA) of SCLC, EAC and ESCC groups.

2 Volcano plots of SCEC, SCLC, EAC and ESCC groups.

3 Functional annotation of SCEC, SCLC, EAC and ESCC groups for Gene Ontology (GO).

4 Gene regulatory network plotted by the top 120 DEGs (ranked by FDR) of SCEC, SCLC, EAC and ESCC groups.

**LISTS OF TABLES**

1 Primers used in the qRT-PCR validation of gene expression microarray data.

2 DAVID annotation of DEGs in SCLC group.

3 DAVID annotation of DEGs in ESCC group.

4 DAVID annotation of DEGs in EAC group.

5 DAVID annotation of DEGs co-up regulated in SCEC and SCLC groups.

6 List of regulatory network genes in SCEC group

7 DAVID annotation of regulatory network genes in SCEC group

**LISTS OF FIGURES**

**
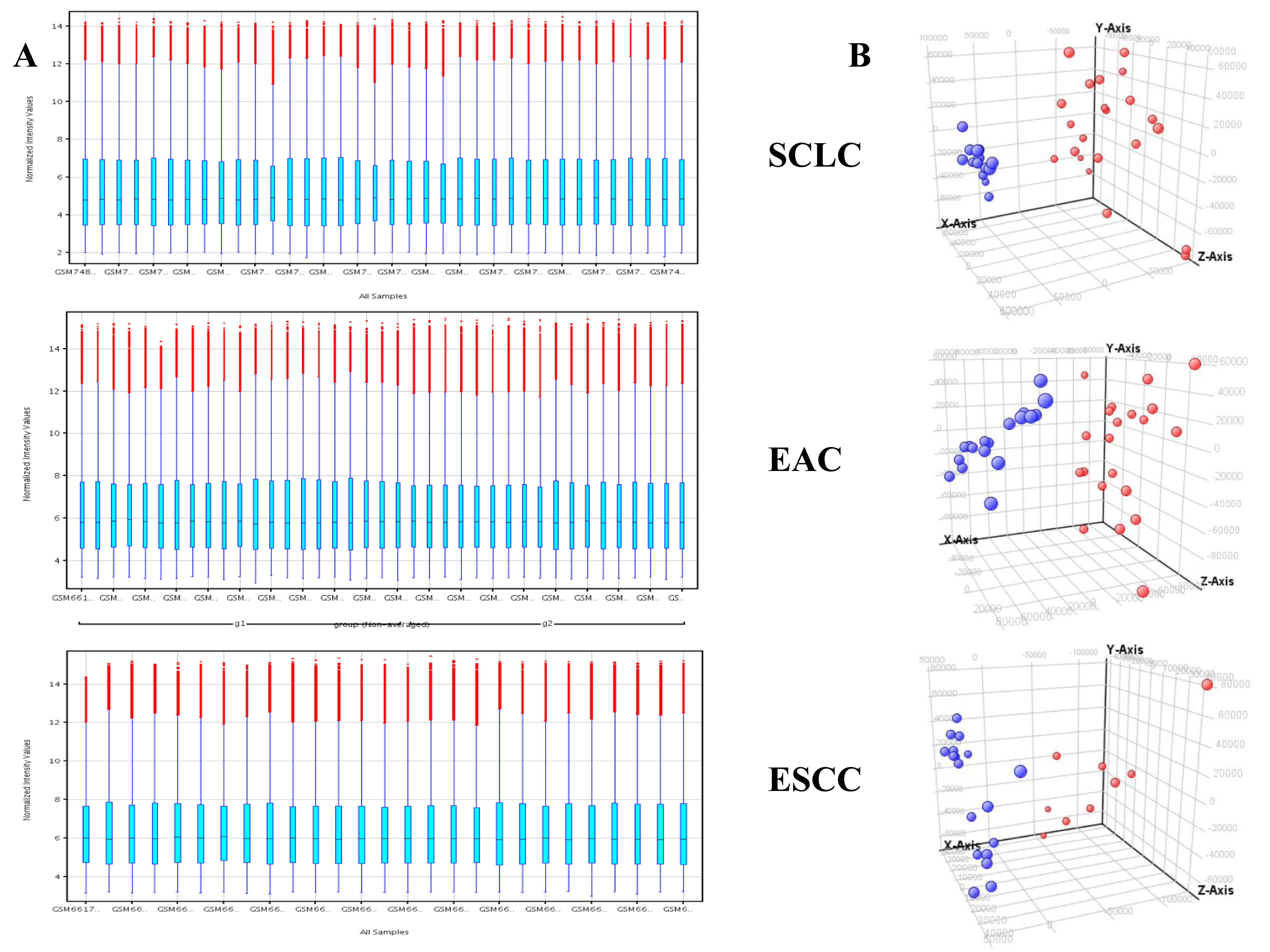
**

**FIGURE S1: Box plot (A) and PCA (B) of SCLC, EAC and ESCC groups.** Box plot and PCA was applied to 3 pairs of SCEC tissues (red) and matched adjacent normal tissues (blue) characterized by the gene expression of all probes on Affymetrix HG U133 Plus 2.0 Array.

**
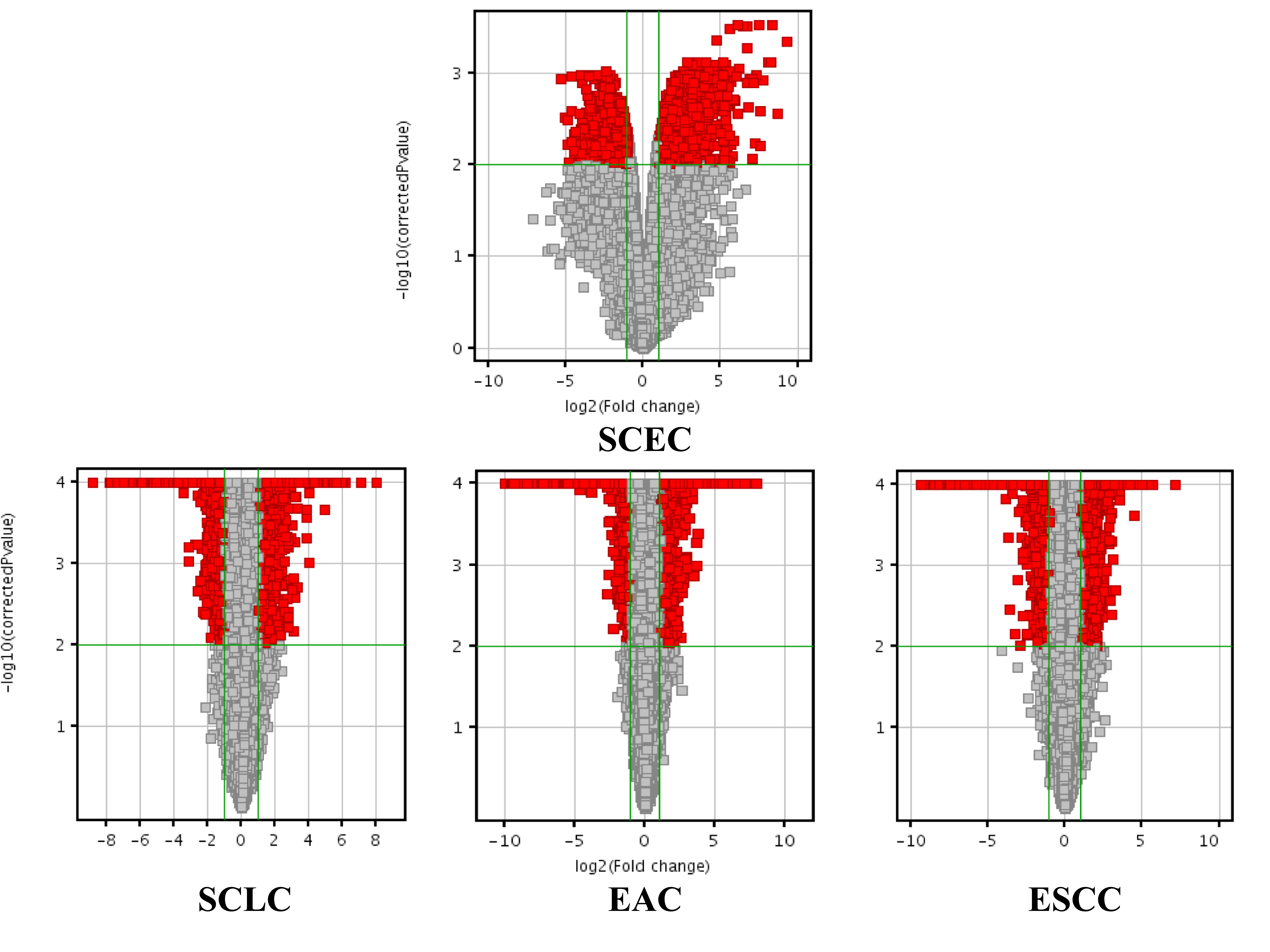
**

**FIGURE S2: Volcano plots of probe sets differing between SCEC, SCLC, EAC/ESCC and matched normal tissues.** Fold change (X axis) is plotted against statistical significance (Y axis) for each probe sets. Genes altered with a fold change ≥2 and FDR <0.01 are depicted in red. Grey represents genes in the arrays that were not found to differ significantly between cancerous samples and matched normal samples.

**
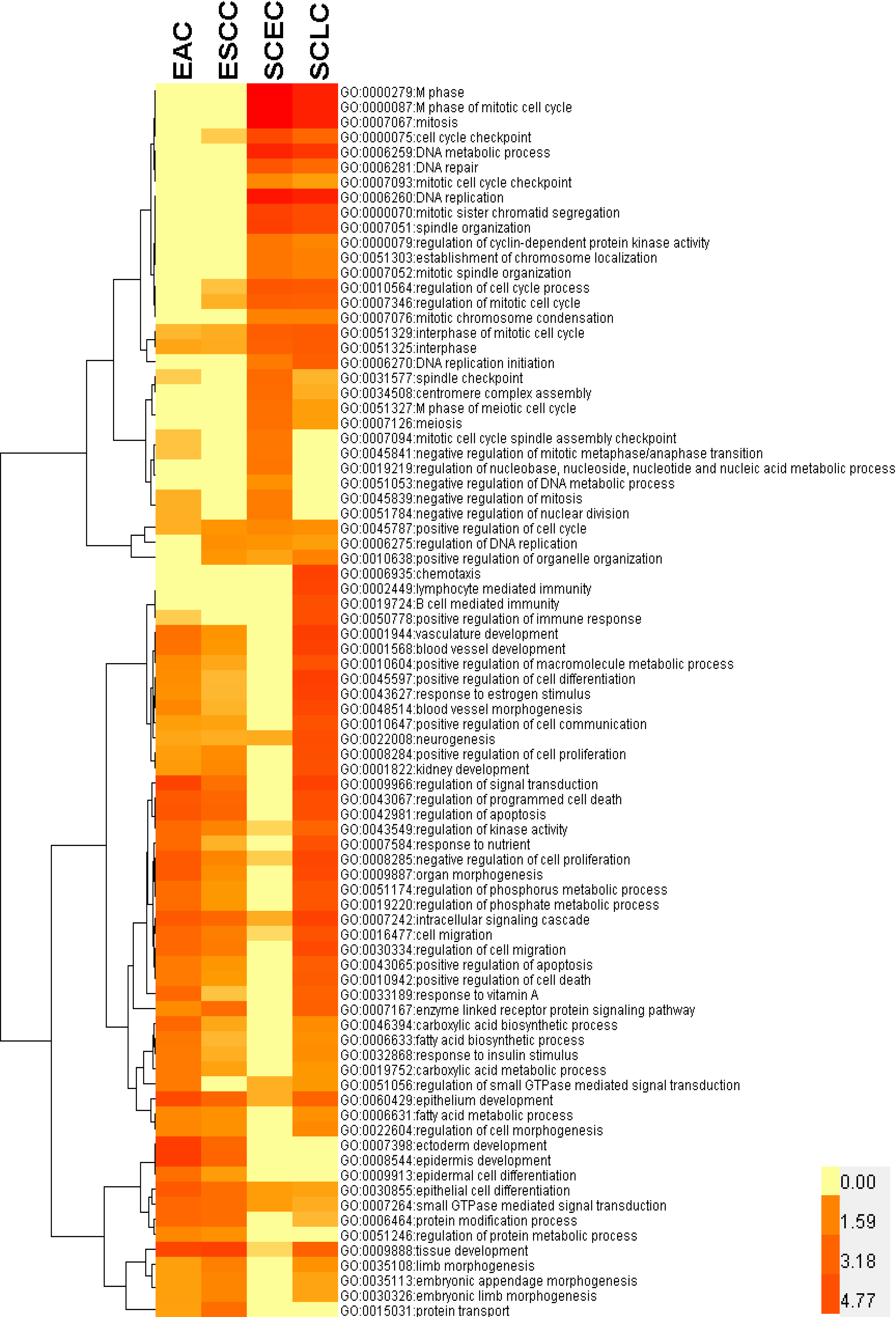
**

**FIGURE S3: Analysis of functional annotation of SCEC, SCLC, EAC and ESCC groups.** Heat map showing analysis of functional annotation results for Gene Ontology (GO) biological process (bp).

**
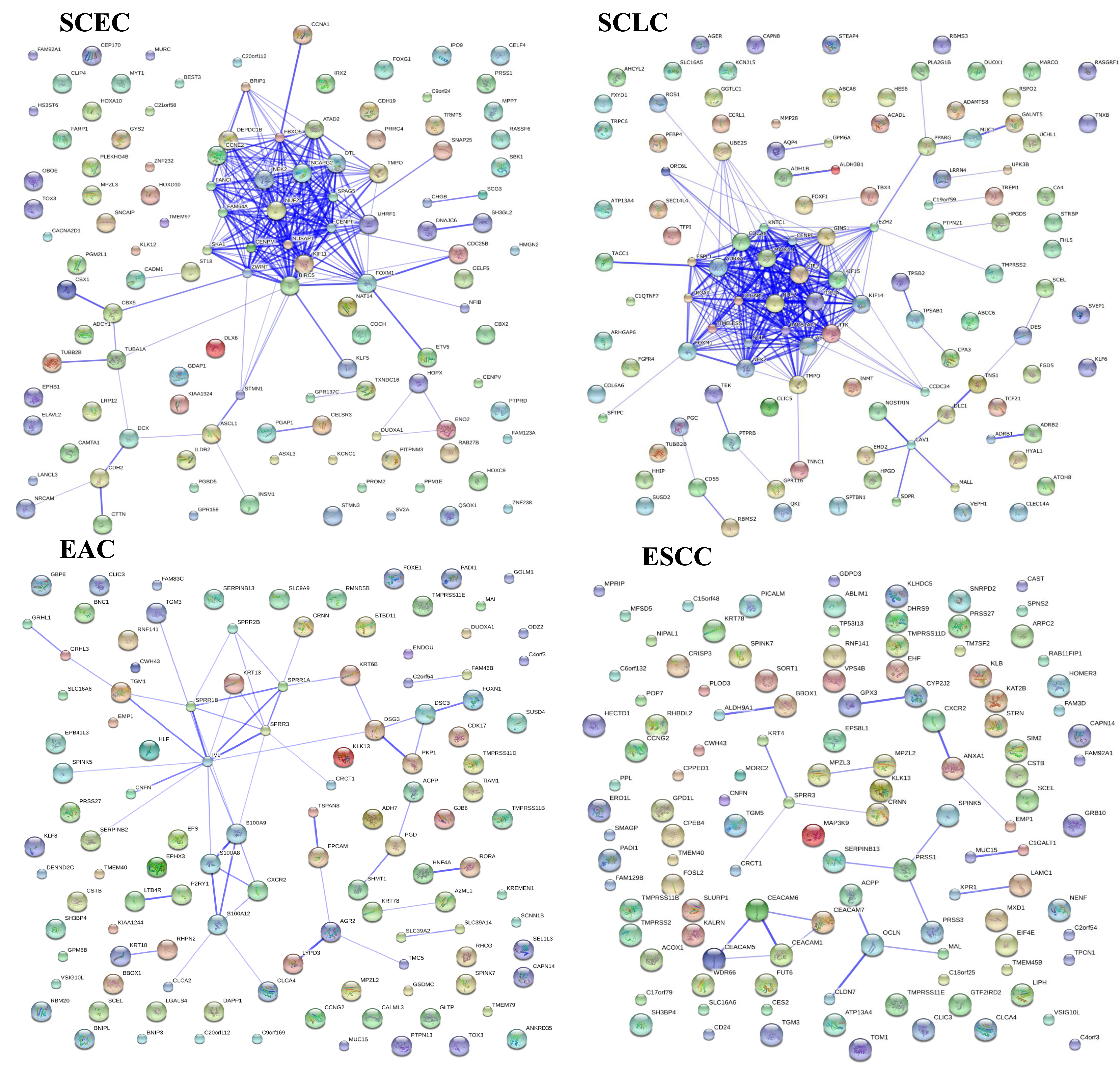
**

**FIGURE S4:** Gene regulatory network plotted by the top 120 DEGs (ranked by FDR) of SCEC, SCLC, EAC and ESCC groups.

**LISTS OF TABLES**

**TABLE S1 Primers used in qRT-PCR for microarray gene expression validation**

| **Gene** | **Forward Primer (5’-3’)** | **Reverse Primer (5’-3’)** | **Product** |
| --- | --- | --- | --- |
| β-actin | AAGGTGACAGCAGTCGGTT | TGTGTGGACTTGGGAGAGG | 195bp |
| INSM1 | GTATTCGCTGTGTTCATGGTC | CGCTACATACATAGAGAGCAGAG | 79bp |
| ASCL1 | AACTCCCATCACCTCTAACA | TGAGACGAAAGACACCAACT | 120bp |
| NRCAM | GATGGCGAAGAATGAAGTT | ACAGTGAGGGATAAGGTGTG | 141bp |
| NUF2 | ATGATGCCAGTGAACTCTGAA | GACTTGTCCGTTTTGCTTTTG | 160bp |
| SNAP25 | CCTGGATATGGGCAATGAGAT | ACACGGGTGGGCACACTTA | 146bp |
| PTP4A3 | GCTTCCTCATCACCCACAA | CCGTACTTCTTCAGGTCCTCA | 70bp |
| RFC4 | CACCCGATTCTGTCTTATCTG | TCCTTCTTGGCAATGTCTAGT | 135bp |
| REST | TTTAAGAGAAGAGGCATCAG | GATATTAGCAGCAAGACCAG | 127bp |
| APEH | GTATTACCGTGCCCTCAAGAC | AAGTGTGTGCGTAGCCAGA | 135bp |
| FBLN2 | AGACCCCAACTCTGTCCATTC | CTCCAGGCACTCGTCATTGT | 121bp |

**TABLE S2 DAVID annotation of DEGs in SCLC group**

| **Database** | **Name** | **Count** | **Benjamini *p* value** |
| --- | --- | --- | --- |
| KEGG | **Cell cycle**  **DNA replication**  Complement and coagulation cascades  Cell adhesion molecules (CAMs)  Cytokine-cytokine receptor interaction  Focal adhesion  Pathways in cancer  ECM-receptor interaction | 71  24  38  60  102  82  119  39 | 1.28E-09  2.70E-04  2.80E-04  4.72E-04  0.00110  9.42E-04  0.00584  0.00662 |
| REACTOME | **Cell Cycle, Mitotic**  Signaling by Rho GTPases  Integrin cell surface interactions  Hemostasis  **DNA Replication**  **DNA Repair** | 148  58  40  91  43  40 | 2.52E-15  7.99E-05  7.56E-04  0.00106  0.00392  0.0472 |
| GO BP  (TOP10) | **M phase**  **DNA replication**  **Mitosis**  **M phase of mitotic cell cycle**  **DNA metabolic process**  Positive regulation of cell differentiation  Vasculature development  Blood vessel development  Regulation of signal transduction  Response to estrogen stimulus | 154  103  114  115  196  103  110  107  301  57 | 4.52E-14  3.62E-14  3.59E-14  8.92E-14  7.21E-09  6.35E-08  8.13E-08  1.64E-07  1.94E-07  1.97E-07 |
| GO MF | Protein kinase activity  Heparin binding  **Adenyl ribonucleotide binding**  Cytokine receptor activity  **Guanyl ribonucleotide binding**  Double-stranded DNA binding  **Pyrophosphatase activity** | 199  47  438  27  124  40  227 | 0.00241  0.00239  0.00280  0.0356  0.0614  0.0548  0.0472 |

^a^ Threshold values: count ≥10 and Benjamini p value <0.01.

^b^ The biological processes or pathways in common between SCEC and SCLC were in bold.

*Abbreviations:* GO= gene ontology; BP= biological process; MF= molecular function.

**TABLE S3 DAVID annotation of DEGs in EAC group**

| **Database** | **Name** | **Count** | **Benjamini *p* value** |
| --- | --- | --- | --- |
| KEGG | Endocytosis  **ECM-receptor interaction**  **Axon guidance**  **Adherens junction**  **Pathways in cancer**  Alanine, aspartate and glutamate metabolism | 64  34  47  31  96  15 | 0.0322  0.0355  0.02591  0.0375  0.0386  0.0425 |
| REACTOME | Metabolism of amino acids  Signaling by Rho GTPases  **Signaling by PDGF**  Metabolism of lipids and lipoproteins | 53  41  25  44 | 0.00630  0.0133  0.0141  0.0421 |
| PANTHER | **Integrin signalling pathway** | 75 | 0.0483 |
| GO BP  (TOP10) | **Ectoderm development**  **Epidermis development**  Regulation of signal transduction  **Tissue development**  **Epithelium development**  Regulation of apoptosis  Negative regulation of cell proliferation  **Intracellular signaling cascade**  **Regulation of programmed cell death**  Organ morphogenesis | 85  80  263  207  87  233  119  340  233  171 | 5.58E-08  4.01E-08  4.85E-07  1.05E-06  4.14E-06  5.81E-05  1.03E-04  1.01E-04  9.08E-05  9.34E-05 |
| GO MF | **Guanyl ribonucleotide binding**  Ras GTPase activator activity  Steroid hormone receptor activity | 116  37  23 | 0.0137  0.0157  0.0343 |

^a^ Threshold values: count ≥10 and Benjamini p value <0.01.

^b^ The biological processes or pathways in common between EAC and ESCC were in bold.

*Abbreviations:* GO= gene ontology; BP= biological process; MF= molecular function.

**TABLE S4 DAVID annotation of DEGs in ESCC group**

| **Database** | **Name** | **Count** | **Benjamini *p* value** |
| --- | --- | --- | --- |
| KEGG | **ECM-receptor interaction**  Focal adhesion  Valine, leucine and isoleucine degradation  Tight junction  **Fatty acid metabolism**  **Pathways in cancer**  Adherens junction  **Axon guidance** | 42  74  24  48  20  94  29  43 | 3.57E-06  5.13E-05  3.83E-04  0.00742  0.00790  0.0397  0.0468  0.0447 |
| REACTOME | **Signaling by PDGF**  **Axon guidance**  Integrin cell surface interactions | 31  23  28 | 2.14E-05  9.81E-04  0.0330 |
| PANTHER | Integrin signalling pathway | 85 | 2.10E-05 |
| GO BP  (TOP10) | **Tissue development**  **Epidermis development**  **Regulation of apoptosis**  **Ectoderm development**  **Regulation of programmed cell death**  Rpithelium development  **Intracellular signaling cascade**  Enzyme linked receptor protein signaling pathway  Protein modification process  Epithelial cell differentiation | 201  66  216  69  216  76  314  103  354  50 | 2.96E-07  9.75E-04  8.30E-04  0.00108  0.00107  9.21E-04  0.00135  0.00230  0.00287  0.00299 |
| GO MF | **Guanyl ribonucleotide binding** | 105 | 0.0490 |

^a^ Threshold values: count ≥10 and Benjamini p value <0.01.

^b^ The biological processes or pathways in common between EAC and ESCC were in bold.

*Abbreviations:* GO= gene ontology; BP= biological process; MF= molecular function.

**TABLE S5 DAVID annotation of DEGs co-up regulated in SCEC and SCLC groups**

| **Database** | **Name** | **Count** | **Benjamini *p* value** |
| --- | --- | --- | --- |
| KEGG | **Cell cycle**  **DNA replication**  Base excision repair  Oocyte meiosis  **p53 signaling pathway**  **Progesterone-mediated oocyte maturation** | 28  17  10  14  10  10 | 4.58E-17  1.50E-15  4.55E-05  2.66E-04  0.00361  0.00310 |
| REACTOME | **Cell Cycle, Mitotic**  **DNA Replication**  **Cell Cycle Checkpoints**  **DNA Repair**  **Telomere Maintenance** | 83  27  23  17  12 | 7.36E-52  4.70E-14  4.61E-09  2.84E-05  1.04E-04 |
| BBID | RBphosphoE2F | 10 | 0.00584 |
| GO BP  (TOP10) | **M phase**  **M phase of mitotic cell cycle**  **Mitosis**  **DNA metabolic process**  **DNA replication**  Spindle organization  **DNA repair**  Mitotic sister chromatid segregation  Cell cycle checkpoint  Regulation of cell cycle process | 85  67  66  83  53  20  42  17  23  23 | 1.10E-46  1.69E-40  4.24E-40  2.27E-30  5.47E-30  2.11E-14  1.41E-12  2.24E-12  5.70E-11  6.29E-09 |
| GO MF | **Pyrophosphatase activity**  **Adenyl ribonucleotide binding** | 48  76 | 3.15E-05  1.98E-05 |

^a^ Threshold values: count ≥10 and Benjamini p value <0.05.

^b^ DAVID annotation results of DEGs co-up regulated in SCEC and SCLC groups were highly concordant with the biological processes or pathways overlapped in SCEC and SCLC groups annotated by DAVID respectively (in bold).

*Abbreviations:* GO= gene ontology; BP= biological process; MF= molecular function.

**TABLE S6 List of regulatory network genes in SCEC group**

| **Gene symbol** | **Gene title** | **Probe set** | **FC** | ***p* value** | **FDR** |
| --- | --- | --- | --- | --- | --- |
| *INSM1* | Insulinoma-associated 1 | 206502_s_at | 321 | 1.60E-08 | 2.91E-04 |
| *ASCL1* | Achaete-scute complex homolog 1 | 209988_s_at | 619 | 7.48E-08 | 4.55E-04 |
| *TUBB2B* | Tubulin, beta 2B class IIb | 214023_x_at | 102 | 9.67E-08 | 5.29E-04 |
| *NUSAP1* | Nucleolar and spindle associated protein 1 | 219978_s_at | 13.1 | 2.14E-07 | 7.65E-04 |
| *DCX* | Doublecortin | 204851_s_at | 261 | 2.47E-07 | 7.65E-04 |
| *NRCAM* | Neuronal cell adhesion molecule | 204105_s_at | 24.8 | 3.26E-07 | 8.11E-04 |
| *SNAP25* | Synaptosomal-associated protein | 202508_s_at | 74.7 | 3.79E-07 | 9.01E-04 |
| *CCNE2* | Cyclin E2 | 205034_at | 13.0 | 4.46E-07 | 9.39E-04 |
| *CENPF* | Centromere protein F | 207331_at | 7.44 | 5.25E-07 | 9.49E-04 |
| *CCNA1* | Cyclin A1 | 205899_at | 17.3 | 4.98E-07 | 9.49E-04 |
| *TUBA1A* | Tubulin, alpha 1a | 209118_s_at | 6.12 | 5.38E-07 | 9.49E-04 |
| *NUF2* | NDC80 kinetochore complex component, homolog | 223381_at | 11.7 | 1.38E-06 | 1.06E-03 |
| *FAM64A* | Family with sequence similarity 64, member A | 221591_s_at | 9.13 | 1.30E-06 | 1.06E-03 |
| *NEK2* | NIMA (never in mitosis gene a)-related kinase 2 | 204641_at | 10.1 | 9.34E-07 | 1.06E-03 |
| *SKA1* | Spindle and kinetochore associated complex subunit 1 | 217640_x_at | 5.34 | 6.67E-07 | 1.06E-03 |
| *SPAG5* | Sperm associated antigen 5 | 203145_at | 9.23 | 6.83E-07 | 1.06E-03 |
| *TMPO* | Thymopoietin | 209754_s_at | 8.36 | 9.86E-07 | 1.06E-03 |
| *CDC25B* | Cell division cycle 25 homolog B | 201853_s_at | 5.53 | 1.16E-06 | 1.06E-03 |
| *NFIB* | Nuclear factor I/B | 209289_at | 4.76 | 1.18E-06 | 1.06E-03 |
| *CBX1* | Chromobox homolog 1 | 201518_at | 4.28 | 1.46E-06 | 1.06E-03 |
| *CDH2* | Cadherin 2, type 1, N-cadherin | 203440_at | 50.0 | 1.54E-06 | 1.07E-03 |
| *ETV5* | Ets variant 5 | 203348_s_at | 4.93 | 1.61E-06 | 1.07E-03 |
| *FBXO5* | F-box protein 5 | 218875_s_at | 8.89 | 1.65E-06 | 1.07E-03 |
| *DTL* | Denticleless E3 ubiquitin protein ligase homolog | 222680_s_at | 10.6 | 1.63E-06 | 1.07E-03 |
| *KIF11* | Kinesin family member 11 | 204444_at | 6.79 | 1.79E-06 | 1.07E-03 |
| *KLF5* | Kruppel-like factor 5 | 209211_at | -4.07 | 1.97E-06 | 1.13E-03 |
| *FANCI* | Fanconi anemia, complementation group I | 213007_at | 5.58 | 1.98E-06 | 1.13E-03 |
| *STMN1* | Stathmin 1 | 200783_s_at | 11.7 | 2.59E-06 | 1.15E-03 |
| *CTTN* | Cortactin | 214073_at | -5.24 | 2.29E-06 | 1.15E-03 |
| *FOXM1* | Forkhead box M1 | 202580_x_at | 8.26 | 2.47E-06 | 1.15E-03 |
| *ZWINT* | ZW10 interactor | 204026_s_at | 6.47 | 2.68E-06 | 1.16E-03 |
| *CBX5* | Chromobox homolog 5 | 231862_at | 4.95 | 2.73E-06 | 1.17E-03 |
| *CENPM* | Centromere protein M | 218741_at | 4.58 | 2.88E-06 | 1.20E-03 |
| *NCAPG2* | Non-SMC condensin II complex, subunit G2 | 219588_s_at | 5.19 | 3.01E-06 | 1.21E-03 |
| *ATAD2* | ATPase family, AAA domain containing 2 | 228401_at | 6.57 | 3.11E-06 | 1.21E-03 |
| *UHRF1* | Ubiquitin-like with PHD and ring finger domains 1 | 225655_at | 10.7 | 3.22E-06 | 1.23E-03 |
| *DEPDC1B* | DEP domain containing 1B | 226980_at | 11.9 | 3.31E-06 | 1.24E-03 |
| *BRIP1* | BRCA1 interacting protein C-terminal helicase 1 | 235609_at | 7.85 | 3.56E-06 | 1.25E-03 |
| *BIRC5* | Baculoviral IAP repeat containing 5 | 202095_s_at | 7.13 | 3.63E-06 | 1.25E-03 |

*Abbreviations:* FC= fold change; FDR= false discover rate.

**TABLE S7 DAVID annotation of regulatory network genes in SCEC group**

| **Database** | **Name** | **Count** | **Benjamini *p* value** |
| --- | --- | --- | --- |
| REACTOME | Cell Cycle, Mitotic | 12 | 3.47E-36 |
| GO BP5 | Mitosis | 14 | 3.82E-12 |
|  | M phase of mitotic cell cycle | 14 | 2.42E-12 |
|  | M phase | 15 | 9.29E-12 |
|  | Spindle organization | 5 | 5.12E-04 |
|  | Regulation of mitotic cell cycle | 6 | 0.00352 |
|  | Cell cycle checkpoint | 5 | 0.00556 |
| GO MF5 | Tubulin binding | 5 | 0.00731 |
|  | Microtubule binding | 5 | 0.0287 |
|  | Cytoskeletal protein binding | 7 | 0.0262 |

*Abbreviations:* GO= gene ontology; BP= biological process; MF= molecular function.
